# Driving forces for changes in geographical distribution of *Ixodes ricinus* ticks in Europe

**DOI:** 10.1186/1756-3305-6-1

**Published:** 2013-01-02

**Authors:** Jolyon M Medlock, Kayleigh M Hansford, Antra Bormane, Marketa Derdakova, Agustín Estrada-Peña, Jean-Claude George, Irina Golovljova, Thomas GT Jaenson, Jens-Kjeld Jensen, Per M Jensen, Maria Kazimirova, José A Oteo, Anna Papa, Kurt Pfister, Olivier Plantard, Sarah E Randolph, Annapaola Rizzoli, Maria Margarida Santos-Silva, Hein Sprong, Laurence Vial, Guy Hendrickx, Herve Zeller, Wim Van Bortel

**Affiliations:** 1 Medical Entomology Group, MRA, Emergency Response Department, Health Protection Agency, Salisbury, UK; 2 Centre for Disease Prevention and Control, Riga, Latvia; 3 Institute of Parasitology, Slovak Academy of Sciences, Kosice, Slovakia; 4 University of Zaragoza, Zaragoza, Spain; 5 Rue de la Voie Sacrée, Souilly, France; 6 Department of Virology, National Institute for Health Development, Tallinn, Estonia; 7 University of Uppsala, Uppsala, Sweden; 8 , Nolsoy, Faroe Islands; 9 University of Copenhagen, Copenhagen, Denmark; 10 Institute of Zoology, Slovak Academy of Sciences, Bratislava, Slovakia; 11 Hospital San Pedro - Centro de Investigación Biomédica de La Rioja, Logroño, Spain; 12 Aristotle University of Thessaloniki, Thessaloniki, Greece; 13 Ludwig-Maximilians-University Munich, Munich, Germany; 14 Institut National de la Recherche Agronomique, Ecole Nationale Vétérinaire, Agroalimentaire et de l'Alimentation, Nantes, France; 15 University of Oxford, Oxford, UK; 16 Fondazione Edmund Mach, San Michele all’Adige, TN, Italy; 17 Instituto Nacional de Saúde Dr. Ricardo Jorge, CEVDI, Lisboa, Portugal; 18 National Institute of Public Health and Environment (RIVM), Bilthoven, Netherlands; 19 CIRAD, Montpellier, France; 20 Avia-GIS, Zoersel, Belgium; 21 European Centre for Disease Prevention and Control, Stockholm, Sweden

**Keywords:** Tick, Ixodes, Europe, Distribution, Climate, Ecology, Surveillance, Tick-borne disease

## Abstract

Many factors are involved in determining the latitudinal and altitudinal spread of the important tick vector *Ixodes ricinus* (Acari: Ixodidae) in Europe, as well as in changes in the distribution within its prior endemic zones. This paper builds on published literature and unpublished expert opinion from the VBORNET network with the aim of reviewing the evidence for these changes in Europe and discusses the many climatic, ecological, landscape and anthropogenic drivers. These can be divided into those directly related to climatic change, contributing to an expansion in the tick’s geographic range at extremes of altitude in central Europe, and at extremes of latitude in Scandinavia; those related to changes in the distribution of tick hosts, particularly roe deer and other cervids; other ecological changes such as habitat connectivity and changes in land management; and finally, anthropogenically induced changes. These factors are strongly interlinked and often not well quantified. Although a change in climate plays an important role in certain geographic regions, for much of Europe it is non-climatic factors that are becoming increasingly important. How we manage habitats on a landscape scale, and the changes in the distribution and abundance of tick hosts are important considerations during our assessment and management of the public health risks associated with ticks and tick-borne disease issues in 21^st^ century Europe. Better understanding and mapping of the spread of *I. ricinus* (and changes in its abundance) is, however, essential to assess the risk of the spread of infections transmitted by this vector species. Enhanced tick surveillance with harmonized approaches for comparison of data enabling the follow-up of trends at EU level will improve the messages on risk related to tick-borne diseases to policy makers, other stake holders and to the general public.

## Review

### Background

*Ixodes ricinus* (Acari: Ixodidae) is a small hard tick that transmits a large variety of pathogens of medical and veterinary importance. Those commonly reported include *Borrelia burgdorferi s.l.* causing Lyme borreliosis, *Anaplasma phagocytophilum* causing human granulocytic anaplasmosis, *Francisella tularensis* causing tularaemia, *Rickettsia helvetica* and *Rickettsia monacensis* causing spotted fever rickettsiosis, *Babesia divergens* and *Babesia microti* responsible for babesiosis, *Neoehrlichia mikurensis,* tick-borne encephalitis virus, Louping ill virus and Tribec virus.

The most prevalent tick-borne infection of humans in the northern hemisphere is Lyme borreliosis, whose incidence has increased in at least nine European countries over the last decade [1-3]. One plausible cause is the changing geographical distribution, density and activity of the principal vector tick, *I. ricinus,* and/or changed activity that bring people into contact with ticks.

The distribution of *I. ricinus* is known to be changing in Europe, both at extremes of altitude and latitude, as well as within its prior range. The reasons for these changes are manifold, and this paper aims to review the driving forces for changes in the geographical distribution of this tick species in Europe. Before we consider these changes, however, it is useful to summarise the biological and ecological aspects of the life history of *I. ricinus* that underpin the impact of so many different drivers on its survival, seasonal and diel activity and distribution.

Each active life stage attaches to a single host and feeds on blood for a period of days before detaching and then moulting (larvae and nymphs) or producing eggs (female adults) [4]. Larvae do not move horizontally over large distances, so often remain aggregated within their environment whilst waiting for a host. Once they find a host, they may be dispersed by host movement while they feed, before they develop and moult to the nymphal stage; this is repeated at the adult stage. The carriage of feeding ticks, particularly by birds and large mammals, is therefore crucial for the short- and long-range dispersal.

All life stages of *I. ricinus* quest for hosts using an ‘ambush’ technique whereby they climb up vegetation and wait for a host to brush past. During questing, the tick loses moisture so has to descend the vegetation into the litter/mat layer to rehydrate. Moving back into the litter/mat layer reduces the probability of coming into contact with a host and uses up energy stores so is detrimental to tick survival [5]. The suitability of the ground-based vegetation, principally in terms of the degree of moisture it affords, is critical for the ticks’ off-host survival [4,6].

*I. ricinus* possesses light-sensitive cells on the dorsum [7], and also sensory organs, Haller’s organs, at the tip of its appendages. These enable it to detect changes within the environment such as light levels, temperature, carbon dioxide, humidity and vibrations thus indicating the best times to quest and the presence of a host. They feed on a wide range of warm- and cold-blooded vertebrate hosts including small rodents, lizards, passerines, larger mammals such as hedgehogs, hares, squirrels, wild boar, deer and livestock [8-16]. The immature stages of *I. ricinus* are found on hosts of all sizes, from small mammals and birds to ungulates, while adult stages feed more exclusively on larger hosts such as cattle and deer. Larger hosts are therefore essential to maintain tick populations, with populations tending to be lower in the absence of these animals [15,17,18]. The local composition of fauna, and their abundance, greatly affects the numbers of ticks being fed. *I. ricinus* is opportunistic and will therefore feed on humans if the chance arises [19], making these ticks efficient vectors of tick-borne human diseases. Common hosts of *I. ricinus* vary in different geographical regions and habitats and infestation rates vary according to the seasonal pattern of questing activity and host availability.

*I. ricinus* is sensitive to climatic conditions, requiring a relative humidity of at least 80% to survive during its off-host periods, and is therefore restricted to areas of moderate to high rainfall with vegetation that retains a high humidity (i.e. litter layer and soil remain humid during the day). The need for such vegetation, the occurrence of animal hosts for all active stages and the ability to disperse within and between habitats are important pre-requisites for *I. ricinus* survival within a habitat and for the completion of its life cycle [20,21]. Typical habitats vary across Europe, but typically include deciduous and coniferous woodland, heathland, moorland, rough pasture, forests and urban parks.

It follows from the above that climatic changes will have an impact on *I. ricinus* survival, abundance and seasonal activity. *I. ricinus* shows variable degrees of behavioural diapause when overwintering, when questing activity is switched off apparently by shortening day-length in common with other species of this taxonomic group [22] and starts again once temperatures are high enough in the spring [23]. Milder winters/warm springs will allow ticks to quest earlier in the year, but periods of excessive heat and dryness in the spring or summer cause ticks to interrupt their questing [24]. The questing tick population naturally declines later in the summer as ticks find hosts and the next cohort does not appear until the autumn [25]. Warmer summers can allow increased rates of development from one life stage to the next, but the impact at the higher end of natural temperature ranges is less significant than at the lower end (i.e. the temperature-dependent effect is non-linear). Northern temperate tick species are well adapted to survive in sub-zero temperatures, but enhanced snow cover may promote overwintering tick survival by preventing repeat freeze and thaw which may be more detrimental. Colder winters might affect survival of small mammal hosts the following year which could mean fewer blood hosts for ticks [26]. However, enhanced snow cover can also help hibernating small mammals but may not be so good for larger animals such as deer that feed on sprigs of vegetation [27].

*I. ricinus* occurs throughout Europe, west to east from Ireland to the Urals, and north to south from northern Sweden to North Africa [28,29]. Yet, *I. ricinus* ticks continue to be reported in new locations in Europe and there is a perception that its abundance has increased in known endemic areas. This review deals specifically with the known and possible drivers for change in the geographic distribution of *I. ricinus*.

Following expert opinion from tick researchers within VBORNET, a network of entomologist and public health experts supported by the European Centre for Disease Prevention and Control, and a comprehensive literature review [Searches were made of PubMed Google Scholar and Science Direct, with search terms including, *Ixodes ricinus*, expansion, distribution, Europe, with each European country included individually], the risk factors can be divided generally into 1) those directly related to climatic change (acting on the tick, the host or their habitat), 2) those related to changes in the distribution of tick hosts (which may be a direct or indirect effect of human intervention), and 3) other ecological changes (also commonly a direct or indirect effect of human intervention). Drivers of change in distribution are discussed under the following headings: climatic effects at high altitude and latitudes (including impacts of tick habitats and tick hosts), habitat patchiness/connectivity and urban green corridors, expansion of tick hosts, and anthropogenic-specific factors, as well as evidence of expansion to new territories. The challenges in overcoming lack of historical data to assess changes are also discussed.

### Distribution changes of *I. ricinus* at the extremes: high altitude and latitude

#### Evidence of change at altitude

There is a clear biological basis for the reduced abundance of *I. ricinus* with increasing altitude, as documented from vertical transects carried out between 620–1270 m.a.s.l (metres above sea level) during the period 2002–2006 in a mountainous region in Czech Republic [30,31]. The highest altitude where oviposition was recorded was at 1150 m.a.s.l with no difference between egg abundance at different altitudes. The rate of egg survival and hatching, however, decreased with altitude, with no eggs hatching at 1150 m.a.s.l and 33% hatching at 1070 m.a.s.l in 2006. The variability in number of eggs within each batch increased with altitude. The number of ticks moulting from larvae to nymphs and nymphs to adults in the same year as the larval/nymphal blood meal, decreased with increasing altitude. Materna *et al.*[31] suggest that less optimal conditions experienced at higher altitudes may account for this. These “less optimal conditions” must largely, but not exclusively, reflect cooler temperatures with accompanying exponential increase in developmental periods [32] and therefore higher total inter-stadial mortality even if daily mortality rates do not change [23]. There will also be fewer hosts available for ticks at higher altitudes (see below).

Additional effects of microclimate variables and the diversity of ecological factors add to the complexity of the impact that altitude might have on the vertical limit of *I. ricinus* survival. Studies in Switzerland [33-36] have highlighted that the aspect of a mountainside also affects tick abundance, even at the same altitude. *I. ricinus* is reported up to 1450 m.a.s.l in the study sites in Switzerland. However, the density of nymphs differs between south-facing and north-facing slopes. Density decreased with altitude on south-facing slopes [34], but increased with altitude on north-facing slopes [35]. This has been explained by the difference in saturation deficit at the same altitudes due to the microclimate. In real terms, however, there were higher numbers of *I. ricinus* on south-facing slopes [36]. In the UK, studies in south Wales found that during the warmer summer months, questing *I. ricinus* were more common on east and west facing slopes [18], and this may change with the seasons.

The altitudinal limit of *I. ricinus* is clearly dynamic, as altitude acts principally as a determinant of climate, and therefore thresholds vary with latitude across Europe. In Italy, the tick becomes far less abundant above 1300 m.a.s.l [37]. Further north, it occurs up to 1450 m.a.s.l in Switzerland [34], 1560 m.a.s.l in Austria [38], 1080–1270 m.a.s.l in Czech Republic [30] and 700 m.a.s.l in Scotland, UK [39]. At the other extreme, in arid parts of Europe typical of the Mediterranean, for example in Greece, no *I. ricinus* ticks have been found below 600 m.a.s.l [40]. Of the ticks removed from humans in Greece during June-September 2008 in areas of low altitude, only 1% (5/519) were *I. ricinus*[41]. With regard to Spain and the Pyrenees, there is a marked west–east gradient on the French-Spanish border. In the west, due perhaps to the Atlantic influence (i.e. greater humidity), the tick is found at sea level, and up to 2000 m.a.s.l. around 2ºE. Towards the east, which is more exposed to a Mediterranean and drier influence, the tick is present only at high altitudes. At about 1ºE, the tick no longer has permanent populations, and only isolated pockets of ticks can be found at about 1000 m.a.s.l. (Estrada-Peña, personal communication).

There is good field-based evidence of an expansion in the altitudinal range of *I. ricinus* from studies in Bosnia & Herzegovina, the Czech Republic and Slovakia. Evidence from Omeragic [42] suggests that the altitudinal threshold of *I. ricinus* in Bosnia & Herzegovina in the 1950s was <800 m.a.s.l, and this increased to 900 m.a.s.l by the 1960s, and to 1190 m.a.s.l by 2010. Similar historical data also exists for the Czech Republic, from two separate mountain ranges. In Sumava, the highest limit of tick survival in studies in 1957 was 700 m.a.s.l. By the 1990s, ticks were being found up to and above 700 m.a.s.l in areas previously thought to be tick free, and by 2001, this had increased to 1100 m.a.s.l [43]. Similarly in Krkonose, in the 1950s and 1980s the threshold remained at 700–750 m.a.s.l. Despite the absence of any notable change in land use over 50 year period [44], the altitudinal limit increased to 1180 m.a.s.l by 2002 and 1250 m.a.s.l by 2006 [30,45]. In Slovakia, a similar change is also evident with an increase from 800 m.a.s.l to ~1200 m.a.s.l [46]. Although sampling strategies differed between studies within countries, all three examples demonstrate clear evidence of an altitudinal expansion of *I. ricinus*.

#### Climate as driver for altitudinal change

Changes in climate over recent years have been recorded throughout Europe, including at high altitude in the Czech Republic. Daniel et al. [47] suggest that the expansion of *I. ricinus* to higher altitudes was due to increased annual and seasonal temperatures and rainfall, and consequently an extended period to allow for tick development. Danielová *et al.*[44,48] reported that in the Moravian highlands in the Czech Republic, temperatures during spring/summer had increased by an average of 2.8°C since the 1960s, creating conditions at altitude similar to that previously found at lower altitudes, thus possibly making them more suitable for sustaining *I. ricinus* populations. They suggest that increased temperatures during January and February will also influence tick host survival, providing ample small mammal hosts for immature stages. In Krkonose, Danielová *et al.*[49] reported that at 1000 m.a.s.l, the mean annual temperature had increased by 1.4°C between 1961 and 2005, with a 3.5°C increase in temperature during spring and summer.

Increasing temperatures will have a disproportionate effect on tick populations under the cooler and limiting conditions at higher altitudes because of the non-linear positive relationship between rates of development and temperature [32,50]. At the same time, the season for development (i.e. with temperatures above the minimum level for development) [30], and the period permissive for questing tick activity (i.e. above a critical temperature threshold), will be extended. For example, studies in Hungary compared the onset of autumn activity of the tick between the 1950s [51] and 2000s [52], finding that autumn activity started and ended one month later and that tick activity (albeit low) was recorded during the winter months, which was not observed in the earlier study. Climate change is usually considered as a gradual trend over an extended period of time, although in fact throughout the mid latitudes of Europe, most of this increase occurred abruptly in 1989 (see [53] for detailed results for the Baltic States that also apply to all sites between 60 and 42^o^N so far examined – Sarah Randolph unpublished data); large inter-annual differences in the length of the seasons and the abundance of ticks will still be expected.

At high altitudes, snow cover during winter may enhance tick survival by minimizing repeated freezing and thawing in litter layers [27] (conversely it may reduce moisture availability), while warmer summers can contribute to decreased mortality of ticks as a result of faster development times [54]. In more arid parts of Europe, around the Mediterranean, humidity is a controlling factor in the distribution of *I. ricinus*, exemplified by studies in Greece that report a lower altitudinal limit of 600 m.a.s.l. [40,41].

Climate cannot act alone; furthermore, *I. ricinus* cannot physically ascend in altitude without a mode of transport. Clearly such movement is facilitated by hosts carrying ticks to sites that have now become favourable for tick survival [30]. Jaenson *et al.*[55,56] suggest that increased winter mean temperatures at higher altitudes and latitudes and an extended vegetation period (VP) have permitted roe deer (*Capreolus capreolus*) to spread to and inhabit previously inhospitable areas of the Alps and Scandinavia. Such deer movements have allowed *I. ricinus* to be transported northwards on the Scandinavian Peninsula, resulting in a significantly increased tick range during the last 30 years [56]. Climatic changes have also reduced the length of snow cover (which in turn can negatively impact tick survival) in alpine areas previously only utilised by roe deer during the summer period, thus lessening the significant limiting factor to roe deer distribution and permanence at altitude [57]. Furthermore, deer tend to move to higher altitudes in spring thus acting as a mode of expansion for *I. ricinus*[31]. The movement of ticks into new areas does not necessarily result in an established population, but the success rate can be higher in less fragmented habitats with suitable vegetation, climate and host availability [58].

#### Habitat change for ticks and mammal hosts at high altitude

In Italy there have been similar reports of increased temperature at high altitude since the 1980s, but other factors evidently contribute to suitability for *I. ricinus* habitat at high altitude, particularly changes in forest and wildlife management [59]. For example, between 1950 and 2002, forest coverage had increased by 2.2%, the area of coppicing (whereby young tree stems are repeatedly cut down to near ground level to enable harvesting of wood) had decreased by 11.8%, and high stand forest had increased by 10.8%. Furthermore, rather than being managed purely for timber, forests are now considered as more complex ecosystems. All these factors, both singly and in concert, will affect the available habitat for *I. ricinus*. With respect to animals, there has been a significant increase and re-population of the Alps by roe deer since the 1940s [59]. The recent reporting of *I. ricinus* feeding on mammal species that inhabit traditionally unfavourable conditions for ticks (i.e. chamois, *Rupicapra rupicapra*) may suggest the possibility for alternative tick-host interaction cycles (A. Rizzoli personal communication).

Similar habitat and tick-host changes have been reported in Slovakia and the Czech Republic. In Slovakia, there is evidence over the last 20 years that areas of formerly cultivated land at high altitude have been left uncultivated, possibly offering additional habitat both for wild rodents and *I. ricinus* (Maria Kazimirova pers. comm.), with ticks found in new regions of Slovakia where populations of *I. ricinus* were previously unrecorded [60]. In the Czech Republic, the harvesting of deer populations at high altitude has increased due to their increased abundance [61]. This increase in host availability may have contributed to the increase in *I. ricinus* at these altitudes. Furthermore, at newly colonised sites for ticks, the local small mammal population would assist in sustaining immature *I. ricinus* stages.

#### Climatic effects at high latitude

With regard to the northern extent of the range of this tick, *I. ricinus* occurs in northern parts of the UK [62] and Denmark (both restricted by the North Sea), and in northern parts of Poland and the Baltic States (restricted by the Baltic Sea). Therefore latitudinal expansion is only an important issue in Fennoscandia because it extends much further north than the rest of Europe.

Various studies have monitored and explained the northward spread of *I. ricinus* in Sweden [55,56,63-66]. Field data and a questionnaire study revealed a shift in distribution during the 1980s and 1990s [64]. The shift was linked climatically to a reduction in the number of days below −12°C during winter, as well as generally mild winters and extended spring and autumn seasons. During this time there was no apparent change in land use that could explain such a tick expansion, but an increase in roe deer numbers was generally accepted and a recent review has argued convincingly that the expansion of the roe deer population and the warmer climate are the two main factors permitting *I. ricinus* to spread so rapidly and extensively to many previously tick-free localities of Northern Sweden [56].

A generally warmer climate can exert an effect in ways already outlined above with respect to warming at high altitudes. *I. ricinus* was commonly encountered in areas where the VP exceeded 180 days, but rarely where it remained below 160 days. There was also a reduced duration of snow cover. With fewer than 125 days of snow cover, *I. ricinus* was consistently present, but above 175 days of snow cover it was consistently absent [66]. A prolonged VP and mild winters also favours better survival, increased abundance and higher densities of deer (specifically roe deer) – the main host for the adult ticks. Furthermore, an outbreak of sarcoptic mange during the 1970s drastically reduced the population of foxes, allowing a dramatic increase in deer whose young were no longer preyed upon by foxes; despite a decline in roe deer numbers once the predator populations recovered during the 1990s, deer density remained much higher than before, which would have been favourable for tick populations [56,65].

Similar sorts of abiotic and biotic factors may also apply to Norway, although the driving forces behind an observed 400km northward expansion of *I. ricinus* are not as clearly defined and need further study [67]. Various predictions for the future suggest that *I. ricinus* distribution in Fennoscandia will expand as winter seasons become shorter and milder, VP increases and deciduous woodland expands [55,63,64,68,69]. Also, increased and year-round (as opposed to seasonal) activity of *I. ricinus* activity is expected in southernmost regions and abundance is expected to increase in most of the regions by 2071–2100 [55].

### Distribution changes of *I. ricinus* within its prior range: host expansion & habitat change

#### Expansion of tick host populations

The above argument, that deer expansion appears to be a major driving force for *I. ricinus* range expansion in some parts of Europe, is supported by the results of a questionnaire survey in the UK [70] and fieldwork in Denmark. Roe deer numbers are reported to have increased five-fold in Denmark during the period 1941–2000 [71], and deer are now appearing in new areas, which may have resulted in new foci of *I. ricinus*. The reasons for this are largely anecdotal, but are thought to be due to a combination of events: a behavioural adaptation in roe deer in relation to their tolerance of human disturbance, ongoing afforestation of the country by 10-25%, changes in agricultural practice (including the feeding of deer), and a decline of predators (due to scabies in foxes). Similarly, their expansion to all regions may be limited by the occurrence of sandy soils in some regions of the country [72].

Although deer are commonly the most important wild animal in sustaining *I. ricinus* populations, other vertebrate species may play that role in some localities. In certain parts of Germany, where roe deer populations have reportedly remained stable, large increases in populations of wild boar have been linked with increases in *I. ricinus* abundance [16]. There are limited data across Europe though on the role of wild boar as hosts for *I. ricinus*. On some small isolated islands in the Baltic Sea, such as Gotska Sandön and other small islands along the Fennoscandian coastline, cervids are absent, but the *I. ricinus* populations can be maintained by mountain hares (*Lepus timidus*) that are the only host for all active stages of the tick populations [73]. In other areas where cervids are absent, livestock can sustain populations of *I. ricinus*[18].

### *The impact of patchiness, connectivity and urban green corridors*

Habitat structure clearly plays an important part in determining whether *I. ricinus* can survive and spread to new regions. While an extended VP and expansion of deciduous woodland is important in northern latitudes, there are also many afforestation and habitat connectivity initiatives within other parts of Europe, well within the prior range of *I. ricinus*. In Spain, there is evidence that long-term and short-term changes in climate suitability are enabling some areas of the country to become more favourable and others less favourable (possibly due to lower rainfall) (Estrada-Peña, personal communication; Jose' Oteo, personal communication). Habitat configuration, that is the degree of connectivity between habitats, is important in determining tick presence/absence and abundance through its effect on tick hosts [58,74]. With more abundant *I. ricinus* associated with greater connectivity between patches, any reduction in the distance between patches (thus promoting de-fragmentation of habitats) may promote the probability of successful invasion and establishment of *I. ricinus* in new areas [58].

In the UK and in other European countries, agri-environment schemes encourage de-fragmentation of habitats by providing habitat corridors between ‘patches’ of extant habitat. This provides a corridor for animal dispersal, refuges for their browsing and laying up (e.g. by deer) and consequently will affect the dispersal of *I. ricinus*. There appears to have been an expansion in the range of *I. ricinus* in south-west England during the last decade [62]. This may be due to the expansion of roe deer from the south-west, but agri-environment schemes may also have played a role. Field data from the UK (Medlock, unpublished) suggest that *I. ricinus* are exploiting ‘field margins’ (i.e. margins of arable land left for wildlife), although the occurrence of ticks is influenced by the neighbouring habitat (e.g. woodland versus arable), and the local activities/movements and daily resting behaviours of deer. Woodlands are also being managed as mosaic habitats, with active management of track-side grassland (known in the UK as a ride) within forests generating compartmentalised woodland, specifically for the benefit of butterflies. This provides sunny path-side vegetation for a range of animals and plants. Various factors, such as leaf litter, sward height and occurrence of bracken and bramble, along with a favourable aspect (i.e. orientation) appear to favour tick abundance [75,76]. In the UK, urban green corridors are facilitating a movement of deer into urban areas and there have been repeated problems of deer and ticks in residential gardens [62]. Tick population modelling suggests that an increase in deer density from a low initial level will cause greater abundance of host-seeking ticks (i.e. the hazard to humans), while, conversely, the same degree of deer population growth from a high initial level will decrease the abundance of questing ticks as they find hosts more quickly [50]. Nevertheless, in both cases the overall tick population is enhanced, allowing greater pathogen transmission potential. A new problem is how deer can be managed in urban areas, particularly if ticks continue to become an urban nuisance and transmit pathogens.

### Phylogenetic structure of *I. ricinus* in Europe

A further complication to the argument of dispersal and local establishment of *I. ricinus* is whether the species is a generalist, adapted to a very wide range of climate conditions, or exists as a series of dynamic populations in different regions within Europe, adapted to local prevailing conditions. The only phenotypic analysis on several populations of the species involved the use of cuticular hydrocarbon composition to characterize the chemical profile of different specimens [77,78]. Such analysis showed the presence of well-defined clades of ticks according to the main climate traits in Europe. This is not surprising because cuticular hydrocarbons are the main determinants of the water retention by arthropods. Therefore, such findings according to differing climate zones across the continent point to the detection of “phenotypical groups”.

Studies of allozymic data or DNA sequences of *I. ricinus* in Europe lead to contradictory opinions; from the lack of genetic structure of the species in the target area [79,80] to the sex-biased genetic structure of the populations under field conditions [81], to a lack of consistency between the European and the African populations of the tick [82]. Some studies suggest that the genetic structure of the populations of *I. ricinus* is linked to the type of host used, which in turn is an indicator of the type of habitat used by the species [83]. In any case, there is an urgent need to understand the phylogenetic relationships of the tick in the western Palearctic. Such studies may contribute to understanding the evolution rates of the tick as driven by environmental factors, as well as the main factors driving the associations of the tick and the pathogens they transmit.

### Generic anthropogenic factors, including habitat change

In France, *I. ricinus* is present throughout the country except at mid- and high altitude. There have been no specific studies tracking its spread, in common with almost all countries apart from those described above. It is, however, generally acknowledged that there has been an increase in abundance of *I. ricinus* in France (Olivier Plantard, Jean-Claude George personal communications). Natural, social and anthropogenic factors are generally accepted by French tick experts as contributing to a de-stabilisation of the previous status quo that may have led to an increase in tick abundance [84]. Much of the information is anecdotal, however, and data on the distribution of *I. ricinus* in France are too fragmentary to make any firm assessments of species expansion. Similarly, many unsolicited casual reports from members of the general public, comparing the lack of a tick problem when they were young with the need to protect the current generation of children from exposure to ticks during recreation, all point to a more widespread distribution of ticks in the UK, especially in peri-domestic habitats (Sarah Randolph personal communication). This is presumably due to a range of causes, some of which may have an anthropogenic origin

In Portugal, the recent increase in studies on the epidemiology of tick-borne disease has led to a greater awareness of the occurrence and spatial distribution of *I. ricinus*, but this does not allow a quantification of tick expansion, or indeed constitute evidence of species expansion. There are anthropogenic drivers in Portugal, such as changes in land management, changes in pest control strategies, extensive destruction of habitats by fire and an increase in hunting, all of which could contribute to any real change (both increase and decrease) in the abundance and distribution of *I. ricinus*[85]. Coupled to these factors are the re-introduction and increase in roe deer abundance, as well as climatic changes [85]. The latter, involving greater heat and moisture stress on ticks, could lead to a retraction in the latitudinal range of *I. ricinus* to central and northern parts of the country.

In central and eastern European countries, the reform of agricultural practices after the fall of Soviet rule has led to significant changes in land cover and land use, most of which act synergistically to improve the habitat for ticks and increase human contact with ticks. National herds of cattle and sheep have declined (although the goat population has increased), and the area under cultivation of field crops has diminished, both allowing natural regeneration of herbaceous and woody vegetation [86] and re-invasion by rodents, deer and ticks (Sarah Randolph unpublished observations). At the same time, the massive broadcast use of pesticides has stopped, allowing better tick survival [86]. At the same time, forest ownership, whether State or non-State, determines the public’s access to forests and therefore the degree of overlap of tick and human activities, and also forest management practices such as clear-cutting, which changes the habitat for ticks and also for human recreation. These factors were shown to affect the incidence of tick-borne disease in Latvia, but not in a simple manner [87].

### Strategies for monitoring change in distribution of *I. ricinus* – evidence from across Europe

So far we have presented several theories on the driving forces for change in distribution of *I. ricinus*, and in some cases we have field-based evidence for such effects. Often our opinion on whether ticks are increasing in their range is based largely on anecdotal evidence, which is often hard to quantify. Recently published maps from the UK [62] have compared enhanced surveillance data during 2005–2009 with historical data. By the authors’ own admission, these data are not perfect and rely largely on where ticks are submitted to the surveillance scheme. However, repeated new reports from areas where historically there were no reports have been interpreted as evidence of tick spread. The benefit of this system is that it is an economically viable option for all European countries, as the only costs are the time taken to identify specimens and log the data. The scheme clearly needs to be advertised widely to remove geographical bias and to generate enough data to allow sensible conclusions to be drawn. This kind of ‘surveillance’ can also be supported by questionnaire style studies on lay persons’ reports of increases in tick abundance or their spread [70]. A multi-source approach to analyse changes in distribution in Norway involved comparing historical data sources on tick distribution from 1930s and 1980s with various 21^st^ century sources including: incidence data on Lyme borreliosis and bovine babesiosis, and observational data of tick occurrence from veterinary surgeons, from the public via media sources and from cervid hunters [67]. In Portugal the nationwide distribution of *I. ricinus* is known to be patchy, although a Portuguese surveillance programme for ticks and tick-borne diseases of public health concern (REVIVE) was implemented from 2011 [88]. It is expected that this will develop a robust baseline of species distribution to facilitate studies of possible tick expansion.

Reliable, historical tick distribution data are valuable for assessing changes in the populations of *I. ricinus*[29]. Unfortunately, very few European countries have such data. Instead, incidence of tick-bites or tick-borne disease tends to lead to suggestions that tick abundance/distribution is changing. In the Netherlands no historical data are available, but there is evidence of an increase in tick bites amongst humans [1]. Possible contributory factors suggested are an expansion of nature reserves, an increased abundance of wildlife, a reduction of pesticide use in agriculture and forestry, and climate change. However, an increase in tick bites or tick-borne disease cases may be related purely to increased abundance rather than increased spread and often involve other important factors such as human exposure to ticks, as demonstrated in Turkey [89] and in much of Europe [90].

In addition to evidence for the expansion of *I. ricinus* within countries, there is recent evidence of expansion of *I. ricinus* into new off-shore territories. The first record of *I. ricinus* in the Faroe Islands came from a dog in 1999. This was followed by records of a nymph on a wheatear in 2000, an engorged female on a cat in 2004, an engorged larva on a chiffchaff in 2004 and a nymph on a human in 2005 (Jens-Kjeld Jensen personal communication; [91]). Ticks on birds may be linked to arrival of migrants from endogenous tick regions, but ticks on both cats and humans suggest that moulting and possible establishment has taken place. Further studies are ongoing.

For some countries there is no reported evidence of an expansion in *I. ricinus* distribution. In Estonia for example, there have been extensive collections of *I. ricinus* for pathogen detection since the 1980s. Although fluctuations in tick abundance were noted, no specific studies on changing abundance or distribution have been conducted (Irina Golovljova personal communication). In Latvia, *I. ricinus* is considered largely absent from eastern parts of the country (where *I. persulcatus* is more common) and there is no evidence of recent spread (Antra Bormane personal communication). However the above-mentioned landscape changes (both newly re-generated woodlands and within existing forests) in Latvia could continue to affect tick abundance and distribution in the future.

At the European level, VBORNET has started to map the distribution of *I. ricinus* based on the publication records of the last 10 years and will update these maps based on a compilation of existing data from various sources provided and shared by the members of the network. This unique map resource will form the basis for the better documenting the range expansion of *I. ricinus* and other tick species relevant to public health.

## Conclusions

**Figure 1:**
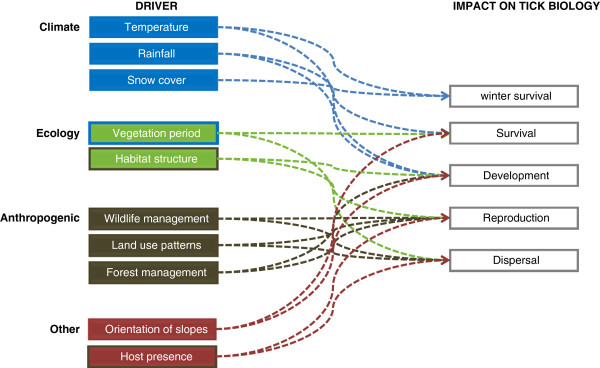
**Conceptual framework of drivers for change in geographical distribution of *Ixodes ricinus*.** The drivers can be divided into those directly related to: climatic change (blue), ecological changes (green), anthropogenic change (brown), and others (red). The colour of the outline indicates the indirect effect of one driver upon the other (see text and Table 1 for details on the mode of actions of the drivers)

Many factors are involved in the latitudinal and altitudinal spread of *I. ricinus* as well as in changes in the distribution within its prior endemic zones. The drivers can be divided into those directly related to climatic change, those related to changes in the distribution of tick hosts particularly roe deer and other cervids, or other ecological changes and anthropogenically induced changes (Table 1, Figure 1). These factors are strongly interlinked and often not well quantified. Better understanding and mapping of the spread of *I. ricinus* (and changes in its abundance) are, however, essential to assess the risk of the spread of infections transmitted by this vector species. Enhanced tick surveillance with harmonised approaches will enable better comparisons to be made of change in trends in distribution at an EU level. This will help improve the messages on the public health risk of tick-borne diseases to policy makers, other stakeholders and to the general public.

**Table 1:** **Overview of the key drivers and their mode of action for change in geographical distribution of *Ixodes ricinus *ticks in Europe**

				**Impact on spread or abundance at**		
**KEY DRIVER**	**Driver**	**Mode of action of driver**	**Impact on tick biology**	**altitude**	**latitude**	**endemic zones**
Climate	Temperature	Increased temperature during winter months	Increased winter survival	X	X	
		Overall increased temperature (winter and summer)	Extended development period	X	X	
	Rainfall	Increased humidity	Extended development period, increased survival			
	Snow cover	insulation, preventing ground temperature falling below 0°C	Increased winter survival	X	X	
	Extended vegetation period (increased temperature and reduced snow cover)	Altitudinal and latitudinal expansion of deciduous woodland creating suitable conditions for *Ixodes ricinus*	Improved microclimate with increased tick survival, and development	X	X	
		Dispersal of roe deer at higher altitude and latitude	Enhanced dispersal and reproduction	X	X	
Anthropogenic	Wildlife management	Increased habitat for *Ixodes ricinus*	Enhanced dispersal	X	X	
		Increased habitat for hosts	Enhanced dispersal	X	X	
		Increased host abundance	Enhanced reproduction			X
	Changes in land use patterns	Increased habitat for *Ixodes ricinus*	Enhanced dispersal			X
		increased habitat for hosts	Enhanced dispersal			X
		Increased host abundance	Enhanced reproduction			X
	Forest management	Reforestation, creation of suitable habitats		X	X	X
Ecological / geographical factors	Habitat structure and connectivity	Increase in suitable environment	Enhanced reproduction and development			X
		Improved hosts dispersal	Enhanced dispersal			X
	Orientation of mountain slopes	Impact on microclimate	Impact on survival, development	X		
	Host dispersal	Behaviour adaptation of roe deer to human presence	Enhanced tick reproduction and dispersal			X

## Competing interests

The author(s) declare that they have no competing interests.

## Authors’ contributions

JMM and KMH conducted the literature review and were responsible with WVB for collating and writing the paper. All other authors provided expert information and comment relevant to their area of expertise and geographic region to ensure that the paper was not geographically biased. The VBORNET project is led by GH, in coordination with WVB and HZ. The work-package responsible for this review is coordinated by JMM. All authors read and approved the final version of the manuscript.
